# How the root bacterial community of *Ficus tikoua* responds to nematode infection: enrichments of nitrogen-fixing and nematode-antagonistic bacteria in the parasitized organs

**DOI:** 10.3389/fpls.2024.1374431

**Published:** 2024-06-28

**Authors:** Xiang-Rui Meng, Yu Gan, Li-Jun Liao, Chao-Nan Li, Rong Wang, Mei Liu, Jun-Yin Deng, Yan Chen

**Affiliations:** ^1^ Ecological Security and Protection Key Laboratory of Sichuan Province, Mianyang Normal University, Mianyang, China; ^2^ Zhejiang Tiantong Forest Ecosystem National Observation and Research Station, School of Ecological and Environmental Sciences, East China Normal University, Shanghai, China

**Keywords:** plant parasitic nematode, bacterial community, assembly process, co-occurrence network, *Ficus tikoua*

## Abstract

Plant-parasitic nematodes (PPNs) are among the most damaging pathogens to host plants. Plants can modulate their associated bacteria to cope with nematode infections. The tritrophic plant–nematode–microbe interactions are highly taxa-dependent, resulting in the effectiveness of nematode agents being variable among different host plants. *Ficus tikoua* is a versatile plant with high application potential for fruits or medicines. In recent years, a few farmers have attempted to cultivate this species in Sichuan, China, where parasitic nematodes are present. We used 16S rRNA genes to explore the effects of nematode parasitism on root-associated bacteria in this species. Our results revealed that nematode infection had effects on both endophytic bacterial communities and rhizosphere communities in *F. tikoua* roots, but on different levels. The species richness increased in the rhizosphere bacterial communities of infected individuals, but the community composition remained similar as compared with that of healthy individuals. Nematode infection induces a deterministic assembly process in the endophytic bacterial communities of parasitized organs. Significant taxonomic and functional changes were observed in the endophytic communities of root knots. These changes were characterized by the enrichment of nitrogen-fixing bacteria, including *Bradyrhizobium*, *Allorhizobium–Neorhizobium–Pararhizobium–Rhizobium*, and nematode-antagonistic bacteria, such as *Pseudonocardia*, *Pseudomonas*, *Steroidobacter*, *Rhizobacter*, and *Ferrovibrio*. Our results would help the understanding of the tritrophic plant–nematode–bacterium interactions in host plants other than dominant crops and vegetables and would provide essential information for successful nematode management when *F. tikoua* were cultivated on large scales.

## 1 Introduction

Plant-associated microbes extend the genetic material of their hosts. The entire genomic material of both the host and its associated microbes should be regarded as an entity, i.e., hologenome (Zilber-Rosenberg and Rosenberg, 2008; Moran and Sloan, 2015; Theis et al., 2016). It was suggested that plant phenotypes were not determined solely by the plant genome but by the entity of hologenome (Zilber-Rosenberg and Rosenberg, 2008; Müller et al., 2016; Trivedi et al., 2020; Angulo et al., 2022). A slight change in root-associated microbes can have substantial effects on host performances (Haney et al., 2015; Hassani et al., 2018). Plants usually modulate their microbial communities to enhance resistance to abiotic and biotic stresses, including plant pathogens (Müller et al., 2016; Trivedi et al., 2020; Angulo et al., 2022).

Plant–parasitic nematodes (PPNs) are among the most damaging pathogens to both crops and natural plants (Jones et al., 2013; Rutter et al., 2022; Yimer et al., 2023). The nematode infections can induce the reorganization of plant-associated microbial communities, which have strong effects on the frequencies and outcomes of PPN parasitism in host plants. The reassembly of rhizosphere and endophytic microbial communities can aggravate or relieve plant damage, but the studies on plant–nematode–microbe interactions have long been concentrated on negative effects. It was suggested that PPNs took on other plant pathogens, inducing synergistic infections when they invaded plant roots (Back et al., 2002; Horiuchi et al., 2005; Escobar et al., 2015). However, more and more studies have demonstrated that these tritrophic interactions can be positive. For example, nematodes can serve as dispersal vectors for rhizobia and facilitate their interaction with legume plants (Horiuchi et al., 2005). Bacteria can help in nutritional supplementations for parasitic nematodes and can restrict the immune response of host plants, which assists the survivor of nematodes (Brown, 2018; Topalović and Vestergård, 2021; Li et al., 2023). Plant-associated microbes can also suppress PPN infection by competing for positions and nutrients, secreting toxic compounds, and/or triggering plant defenses (Schouten, 2016; Topalović and Heuer, 2019; Topalović et al., 2020).

The effects of plant-associated microbes on both PPNs and host plants are highly taxa-dependent. Even different genotypes within a plant species may respond to similar microbial communities differently (Haney et al., 2015; Topalović and Heuer, 2019; Trivedi et al., 2020). These species-dependent interactions have resulted in powerful microbial agents for PPN control that are inefficient in other plants (Topalović and Vestergård, 2021). So far, the studies on the tritrophic plant–nematode–microbe interactions have focused on dominant crops and vegetables (Jones et al., 2013; Wilschut and Geisen, 2020). The responses of other plants were largely unknown, even unaware, leading to a significant underestimation of PPN damages (Jones et al., 2013).

*Ficus tikoua* (*Ficus*, Moraceae) is widely distributed in Southwest China (Deng et al., 2020). This species has versatile application potentials, which can be used as resources for fruits, traditional medicines, and ecological restorations (Wei et al., 2012; Chai et al., 2017). In recent years, a few farmers tried to cultivate this species for commercial purposes, and PPNs are presented in the roots of planting individuals. However, we knew nothing about the response of this species to nematode infection, which would undermine the chance of success in PPN controls. To explore the effects of nematode parasitism on microbial communities associated with *F. tikoua*, we used 16S rRNA sequences to compare the bacterial communities of rhizosphere soils, roots, and root knots associated with healthy and infected individuals, aiming to explore the questions: (1) Are the structures of bacterial communities associated with healthy and infected individuals different? If so, which community, rhizosphere or endophytic bacteria, was affected more by PPN infections? (2) Have the infected plants enriched special bacteria? If so, what is the function of the enriched bacteria? (3) Are assembly processes in the bacterial communities associated with healthy and infected plants different? Our study would help the understanding of the responses of plants other than dominant crops or vegetables to PPN parasitism and also provide information for PPN management when *F. tikoua* were planted on a large scale.

## 2 Materials and methods

### 2.1 Sample collection

Samples were collected from *Ficus tikoua* plants cultivated in the botanical garden of Mianyang Normal University, China (104.35°E, 31.27°N). All plants were cultured under similar conditions. In 2021, root knots indicating nematode infection were observed in some of the mature plants. The presence of nematodes and feeding sites was confirmed through paraffin sections of the root knots (refer to **Supplementary Figure S1**). Furthermore, nematodes were extracted from root knots using the Baermann method (Baermann, 1917), providing additional evidence of nematode parasitism in these plants. In November 2022, a comprehensive inspection of all garden-planted individuals was carried out to differentiate between infected and healthy plants. The soil and leaf nutrients were measured using a stable isotope ratio mass spectrometer. Nematode infection induced a significant decrease in leaf carbon content. No significant changes were observed in soil nutrient contents (**Supplementary Table S1**).

Two groups were defined based on the inspection: the infected group, where individuals showed clear signs of nematode infection resulting in the formation of root knots, and the healthy group, with no visible infection. Five individuals were randomly selected from each group for sampling, ensuring a representative collection of samples for subsequent analysis.

The rhizomes and adhered roots of the selected individuals were carefully cut and pulled out in November 2022. Residual soil particles were gently shaken off. Next, the rhizomes were immediately put into ice boxes and taken back to the laboratory for sample collection. All subsequent sampling procedures were executed on a clean bench. Rhizosphere soils were gently brushed off from the roots of each sampled plant and then sieved through 2 mm mesh sieves. The fine roots (diameter < 2 mm) were collected from healthy plants. Both fine roots and root knots were collected from infected plants. The roots and root knots were surface sterilized using a protocol involving 2.5% NaClO for 10 min, followed by 75% ethanol for 3 min. In total, 10 rhizosphere soil (NRS1–5 for healthy, IRS1–5 for infected plants), 10 roots (NRT1–5 for healthy, IRT1–5 for infected plants), and five root knot samples (IRK1–5) were collected (root knots were only presented in affected plants). All collected samples were frozen in liquid nitrogen for 15 min and then stored at −80°C until further analysis.

### 2.2 DNA extraction and PCR amplification

Total microbial genomic DNA from both rhizosphere soil and endophytic bacteria of *F. tikoua* was extracted using the E.Z.N.A.^®^ soil DNA Kit (Omega Bio-tek, Norcross, GA, USA).

The 338F and 806R primer sets were used for amplifying the V3–V4 region of the bacterial 16S rRNA gene in rhizosphere soil bacteria (Claesson et al., 2010). The reaction mixture for rhizosphere soil bacteria was prepared using the TransGen AP221–02: TransStart Fastpfu DNA Polymerase system. The components of the mixture included 4 μL of 5× FastPfu buffer, 2 μL of 2.5 mM dNTPs, 0.8 μL of each forward and reverse primer at a concentration of 5 μM, 0.4 μL of FastPfu polymerase, and 0.2 μL of BSA. To this, approximately 10 ng of template DNA was added, and the total volume was adjusted to 20 μL with ddH_2_O. This PCR was executed on an ABI GeneAmp^®^ 9700. The PCR amplification began with an initial denaturation at 95°C for 3 min, followed by 13 cycles of 30 s at 95°C, 30 s at 55°C, and 45 s at 72°C. The process concluded with a final extension at 72°C for 10 min, after which the reactions were held at 10°C.

The 799F and 1193R primer sets were utilized for endophytic bacteria, minimizing interference from chloroplast genes (Wang et al., 2018). The Pro Taq system was used to amplify endophytic bacteria. The reaction mixture included 0.8 μL of each forward and reverse primer at a 5-μM concentration, approximately 10 ng of template DNA, 4 μL of 5× FastPfu buffer, 2 μL of 2.5 mM dNTPs, 0.4 μL of FastPfu polymerase, and 0.2 μL of BSA, with ddH_2_O added to reach the final volume of 20 μL. This PCR was performed on an ABI GeneAmp^®^ 9700. The cycling parameters were set to an initial denaturation at 95°C for 3 min, followed by 13 cycles of 30 s at 95°C, 30 s at 55°C, and 45 s at 72°C, and concluding with a final extension at 72°C for 10 min, before being held at 10°C.

### 2.3 Sequencing

The PCR products were purified. Purified amplicons were pooled in equimolar amounts and subjected to paired-end sequencing on an Illumina PE300/PE250 platform (Illumina, San Diego, CA, USA) following the standard protocols provided by Majorbio Bio-Pharm Technology Co. Ltd. (Shanghai, China).

### 2.4 Data processing and analysis

Following demultiplexing, the sequences were subjected to quality control using FASTP (Chen et al., 2018). This process involved filtering out bases with a quality score below 20 at the ends of reads and trimming bases if the average quality score within a 50-bp sliding window fell below 20. Reads shorter than 50 bp postquality control and those containing “N” bases were discarded. Subsequently, the high-quality reads were merged using FLASH (Magoč and Salzberg, 2011). The merged sequences were then denoised using the DADA2 plugin in QIIME2 (Bolyen et al., 2019) under default parameters, resulting in the generation of amplicon sequence variants (ASVs) based on within-sample error profiles. Sequences identified as chloroplast and mitochondrial in origin were excluded from all samples. To mitigate the impact of sequencing depth variability on alpha and beta diversity analyses, sequences from each sample were rarefied to the minimum sample sequence count, achieving an average good coverage of 99.09%.

Taxonomic classification of ASVs was performed using the Naive Bayes consensus taxonomy classifier in QIIME2, referencing the SILVA 16S rRNA database. Metagenomic functions were predicted specifically from 16S rRNA data using FAPROTAX (Sansupa et al., 2021).

α-Diversity and β-diversity analyses of *F. tikoua* samples were conducted using R’s vegan package to explore microbial community structure and variations. For α-diversity, Shannon, Simpson, ACE, and Chao indixes were computed, assessing species richness and diversity. Species abundance variations among different sample groups were assessed at the genus level using Kruskal–Wallis (multiple groups) and Wilcoxon rank-sum tests (pairwise). The false discovery rate (FDR) method was applied for multiple comparison corrections, and confidence intervals were estimated using 1,000 bootstraps. For β-diversity, principal coordinate analysis (PCoA) with Bray–Curtis distance was used to evaluate compositional differences at the genus level among sample groups. Permutational multivariate analysis of variance (PERMANOVA) was employed to test the differences.

To investigate microbial network patterns and their forming factors, the co-occurrence network analysis was performed using R’s “igraph” package (Csardi and Nepusz, 2006). The co-occurrence networks of the endophytic bacterial communities in healthy roots (NRT), affected roots (IRT), and root knots (IRK) were constructed at the genus level independently. Each group (NRT, IRT, and IRK) had five samples. In this analysis, each node represents a genus, with links indicating cooccurrence relationships defined by Spearman’s correlation > 0.7 or ≤ 0.7 (*p* < 0.05). The networks were visualized in Gephi (Bastian et al., 2009). The β-nearest taxon index (βNTI) and RC_bary_ index were also employed to compare the assemblage process in the bacterial communities between groups (NRT vs. IRT, NRT vs. IRK, and IRT vs. IRK). Processes with |βNTI| > 2 are considered deterministic, whereas those with |βNTI| < 2 are deemed stochastic processes. The RC_bary_ index was applied to measure stochastic processes in microbial communities. RC_bary_ > 0.95 indicates dispersal limitation; RC_bary_ ≤ 0.95 suggests homogenizing dispersal; and RC_bary_ in between signifies nondominant processes (Stegen et al., 2013). The βNTI index was computed using both the “phyloseq” and “picante” packages in R (McMurdie and Holmes, 2013). We categorized endophytic bacteria based on their phylogenetic relationships, estimating intersample microbial community compositional changes based on proximate evolutionary distances. The RCbary index was calculated using the method provided by Stegen (Stegen et al., 2013). Phylogenetic trees were constructed using IQ-Tree1.6.12 (Nguyen et al., 2015), and all visualizations were created using the ggplot2 package in R (Wickham, 2011).

## 3 Results

The rhizosphere soils, roots, and root knots were collected from five nematode-infected and five healthy *F. tikoua* plants. In total, 10 soil samples (NRS1–5 for healthy, IRS1–5 for infected plants), 10 root samples (NRT1–5 for healthy, IRT1–5 for infected plants), and five root knots (IRK1–5) were obtained.

Bacterial 16S rRNA genes were sequenced for all samples using the Illumina MiSeq platform, resulting in a total of 1,408,936 reads (soil sample, 576,530 reads with an average length of 417 bp; root and root-knot, 734,905 reads with an average length of 377 bp). After rigorous filtering and quality control processes, the remaining high-quality effective reads were clustered into 8,448 ASVs for each sample.

### 3.1 The effects of nematode parasitism on the composition of bacterial communities in *F. tikoua*

The phylum composition was different between the bacterial communities associated with rhizosphere soil (NRS, IRS) and plant organs (NRT, IRT, IRK). Three phyla (Proteobacteria, Actinobacteria, and Chloroflexi) were dominant in both endophytic and rhizosphere bacterial communities, with Proteobacteria enriched substantially in endophytic bacteria. Two common phyla, Acidobacteria and Bacteroidota, in rhizosphere soils were gradually diluted in the endophytic bacterial communities from healthy to nematode-infected plants (**Supplementary Table S2**).

**Figure 1:**
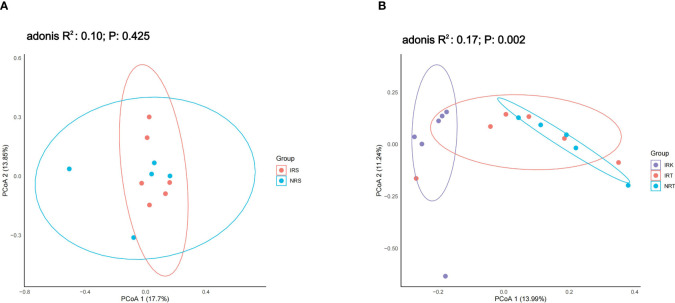
The PCoA results on the genus level. **(A)** The rhizosphere bacterial communities. Sample groups: NRS, rhizosphere soils of healthy plants; IRS, rhizosphere soils of infected plants. **(B)** The endophytic bacterial communities. Sample groups: NRT, roots of healthy plants; IRT, roots of infected plants; IRK, root knots (nematode-parasitized organs).

Nematode parasitism induced significant assemblage shifting in endophytic bacterial communities but not in rhizosphere ones. PCoA analysis could not tell apart the rhizosphere bacterial communities associated with healthy plants (NRS) or with infected plants (IRS) (adonis *R* ^2^ = 0.10, *p* = 0.425), suggesting similar bacterial compositions in the rhizosphere soils of both plant types (**Figure 1A**). However, PCoA revealed significant differences among the endophytic bacterial communities in different sample groups (NRT, IRT, IRK) (adonis *R* ^2^ = 0.17, *p* = 0.002). The IRK samples were totally separated from NRT samples (**Figure 1B**), indicating significant taxonomic changes in the endophytic bacterial communities of nematode-parasitized organs.

Nematode parasitism changed the bacterial diversities in both rhizosphere and endophytic communities. The effects were stronger on endophytic communities than on rhizosphere communities. Higher species richness but similar species diversity occurred in the rhizosphere bacterial communities of infected *F. tikoua* plants (IRS) as compared with those of healthy individuals (NRS). The ACE and Chao indexes of IRS samples were significantly higher than those in NRS samples, while no differences were detected in Shannon and Simpson indexes (**Table 1**). A decreasing trend was presented in the bacterial diversities of endophytic communities from healthy roots (NRT) to infected roots (IRT) and then to parasitized organs (IRK). Significant differences were detected in Shannon, ACE, and Chao indexes between NRT and IRK samples, indicating significantly lower bacterial diversities in root knots than in healthy roots (**Table 1**).

**Table 1:** The alpha diversities of bacterial communities associated with healthy and nematode-infected *F. tikoua* plants.

	Rhizosphere		Endophytic		
	NRS	IRS	NRT	IRT	IRK
Shannon Index (mean ± SD)	7.38 ± 0.12^a^	7.50 ± 0.04^a^	5.29 ± 0.43^a^	5.34 ± 0.27^ab^	4.20 ± 1.20^b^
Simpson Index (mean ± SD)	0.99 ± 0.01^a^	0.99 ± 0.0001^a^	0.97 ± 0.03^a^	0.98 ± 0.01^a^	0.93 ± 0.09^a^
ACE Index (mean ± SD)	3,110 ± 364^b^	3,576 ± 238^a^	914 ± 70^a^	879 ± 51^ab^	635 ± 230^b^
Chao Index (mean ± SD)	3,069 ± 341^b^	3,491 ± 201^a^	910 ± 71^a^	876 ± 51^ab^	630 ± 241^b^

Sample groups: NRS, rhizosphere soils of healthy plants; IRS, rhizosphere soils of infected plants; NRT, roots of healthy plants; IRT, roots of infected plants; IRK, in root knots (nematode-parasitized organs). Different letters indicate significant differences between sample groups within rhizosphere or endophytic communities, respectively. *P* < 0.05.

### 3.2 Assembly process in endophytic bacterial communities in response to nematode parasitism

**Figure 2:**
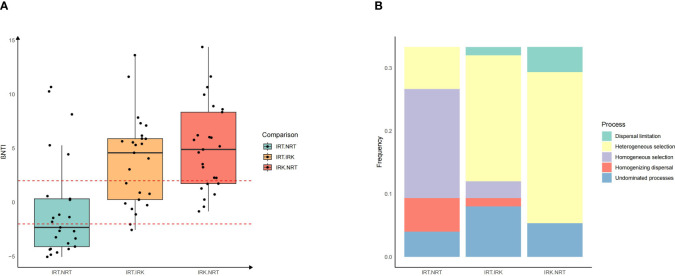
Assembly patterns of endophytic bacterial communities among different sample groups in *F*. *tikoua*. The abbreviations of sample groups are the same as in **Figure 1**. **(A)** βNTI value in each group pair; **(B)** the relative frequencies of different assembly processes in each group pair. The assembly processes are divided into five categories: heterogeneous selection (βNTI > 2), homogeneous selection (βNTI ≤ −2), and dispersal processes (genetic drift, dispersal limitation, processes not dominated) |βNTI| < 2.

βNTI indexes suggested the significant assemblage shifts in the endophytic bacterial communities of nematode-parasitized organs had been shaped mostly by a deterministic process. Pairwise βNTI indexes of IRK-IRT and IRK-NRT were all larger than 2, suggesting heterogeneous selection contributes most to the bacterial taxonomic differences between root-knot samples and other samples (**Figure 2**).

**Figure 3:**
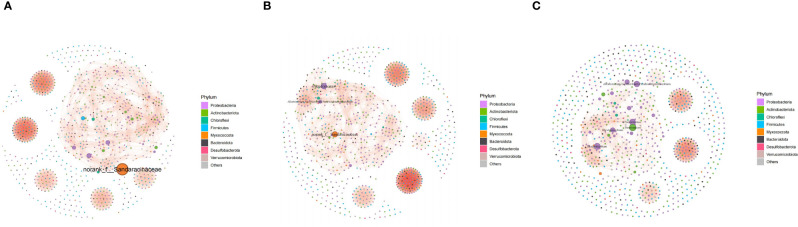
Cooccurrence networks of endophytic bacterial communities associated with healthy and nematode-infected plants of *F*. *tikoua* (*r* ≥ 0.70, *p* < 0.05). **(A)** NRT; **(B)** IRT; **(C)** IRK. In the network, nodes represent genus in the community; all nodes are colored according to bacterial phylum. The abbreviations of sample groups are the same as in **Figure 1** and **Table 1**.

The bacterial cooccurrence network revealed intensive bacterial interaction in endophytic communities in nematode-parasitized organs. The structures were different among the co-occurrence networks of the endophytic communities in different organs. Modularity declined gradually from NRT to IRT and IRK networks. Six connected components were presented in the NRT and IRK networks, while just five components occurred in the IRK network (**Figure 3**; **Supplementary Table S3**). The average clustering coefficient of the IRK network was bigger than that of the NRT network (**Supplementary Table S3**), indicating more intensive bacterial interaction within modules in the IRK network.

**Figure 4:**
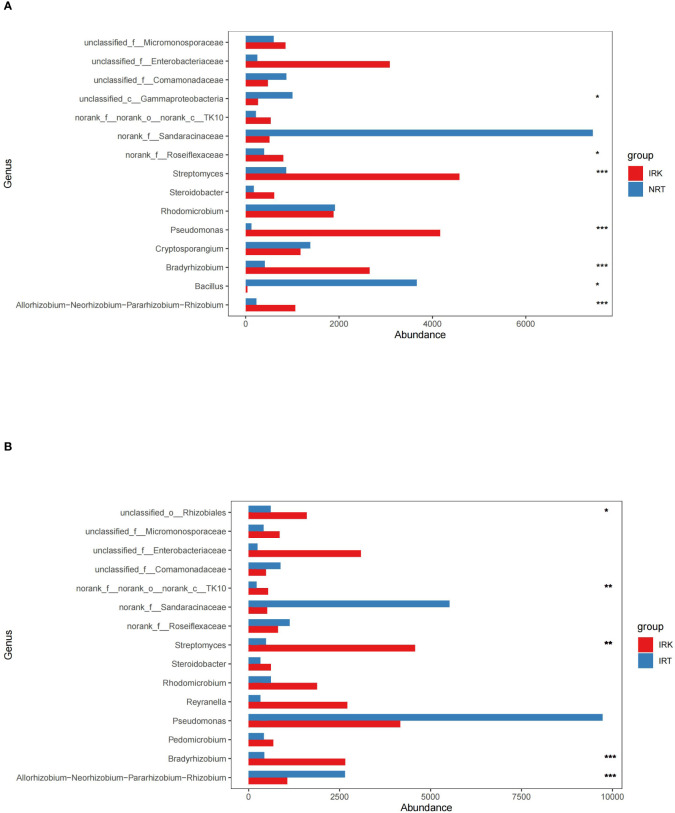
The genus abundance differences in the endophytic bacterial communities of different organ samples. **(A)** IRK&NRT; **(B)** IRK&IRT. The abbreviations of sample groups are the same as in **Figure 1** and **Table 1**. Significant differences are highlighted by an asterisk (^*^ *p* < 0.05; ^**^ *p* < 0.01; ^***^ *p* < 0.001).

Both cooccurrence networks and genus abundance difference tests among the three sample groups detect enrichments of specific endobacteria. The relative frequencies of *Pseudomonas* and *Allorhizobium* increased in IRT networks as compared with NRT networks. Substantial enrichments of *Bradyrhizobium*, *Allorhizobium*, *Streptomyces*, *Pseudomonas*, and *Rhomicrobium* were further presented in the IRK network (**Figure 3**). Genus abundance difference tests also revealed that root knots enriched a series of bacterial genera as compared with both infected or healthy roots, including *Bradyrhizobium*, A*llorhizobiu–Neorhizobium–Pararhizobium–Rhizobium*, *Pseudonocardia*, *Pseudomonas*, *Steroidobacter*, *Rhizobacter*, and *Ferrovibrio* (**Figure 4**).

**Figure 5:**
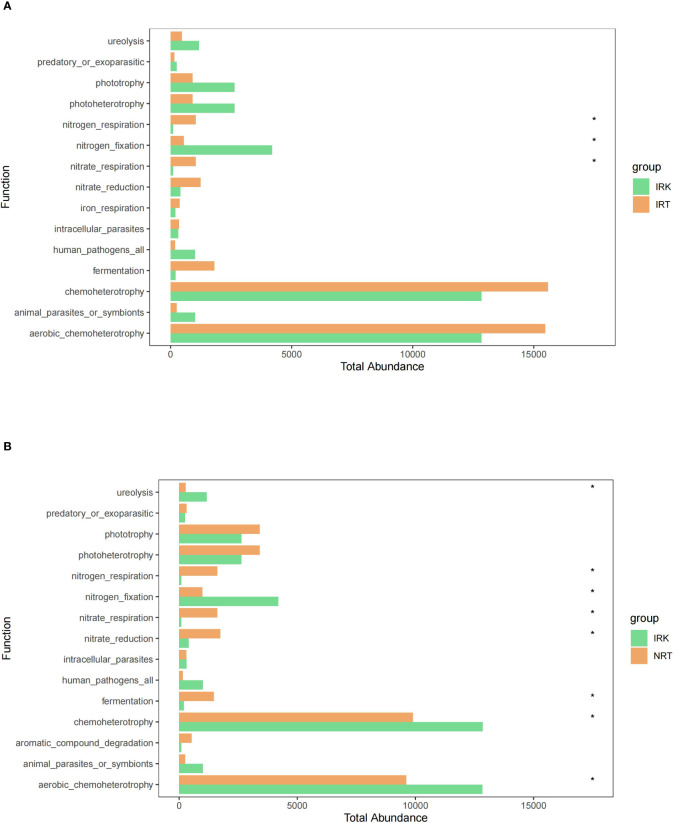
The predicted functional differences in the endophytic bacterial communities of different organ samples. **(A)** IRK&NRT; **(B)** IRK&IRT. The abbreviations of the sample groups are the same as in **Figure 1** and **Table 1**. Significant differences are highlighted by an asterisk (^*^ *p* < 0.05).

FAPROTAX functional annotations and Wilcoxon rank sum tests revealed no functional difference between the NRT and IRT samples. Significant changes were presented in endophytic bacterial communities of root knots as compared with those in either healthy or infected roots. The functional shifts concentrated on the pathways associated with metabolism. Nitrogen fixation was significantly enhanced, while fermentation, nitrogen respiration, and nitrate respiration were significantly weakened in root knots. It is unexpected that no functional changes were detected in the pathways associated with disease resistance (**Figure 5**).

## 4 Discussion

Nematodes are present in the roots of some *F. tikoua* individuals. To explore the effects of nematode parasitism on root-associated bacteria, we compared the rhizosphere and endophytic bacterial communities of nematode-infected and healthy individuals. Nematode parasitism did not affect the rhizosphere community strongly, resulting in an increase in bacterial richness but not diversity in infected individuals. While the endophytic communities had been significantly reshaped by nematode parasitism, significant taxonomic and functional shifts were presented in the endophytic bacterial communities in root knots. The bacteria in relation to nitrogen-fixing and nematode antagonism were enriched.

### 4.1 Nematode infection induced the increase of bacterial richness in the rhizosphere community

The rhizosphere microbial community plays an important role in plant health, providing the frontline defense for plant pathogens (Kerry, 2000; Mendes et al., 2013; Backer et al., 2018). Plants can modulate their associated microbiota when encountering plant pathogens. Documented studies suggested nematode infection might induce significant changes in both the diversities and composition of the rhizosphere microbial community (Topalović et al., 2020; Li et al., 2023). However, nematode infections induced an increase in species richness but not species diversities in the rhizosphere bacterial communities of *F. tikoua*. ACE and Chao indexes were significantly higher in the IRS (healthy individuals) than in the NRS (infected individuals). No significant differences were detected in the Shannon and Simpson indexes between the two sample groups.

Root–microbe interaction is mostly mediated by root exudates (Bais et al., 2006; Pascale et al., 2020). Root exudates provide both resources and chemical signals to mediate the interactions (Bais et al., 2006; Rasmann and Turlings, 2016; Venturi and Keel, 2016). Among them, available C and N resources are critical for microbe activation and growth (Blagodatskaya and Kuzyakov, 2013). It was demonstrated by the ^14^C labeling technique that nematode-infected plants excreted more carbon into soils than healthy plants. Nematode parasitism can induce changes in the quantity and composition of root exudates (Kerry, 2000; Back et al., 2002). Nematode endoparasitism form special feeding sites within host plant roots. These sites serve as resource sinks, drawing nutrients from the host plant and leading to increased leakage of resources into the rhizosphere soils (Kerry, 2000; Bartlem et al., 2014; Siddique and Grundler, 2018). Feeding sites presented in the root knots of *F. tikoua*. It is reasonable to expect more carbon to be supplied into the rhizosphere soils associated with infected plants. Higher available carbon would activate bacteria there, resulting in increased species richness. However, no significant changes were detected in species diversity or composition in the rhizobacteria of infected and healthy plants. It was suggested root exudates usually mediated selective enrichments of special bacteria in the rhizosphere when plants were attacked by pathogens (Bakker et al., 2018; Pascale et al., 2020). Although we did not detect significant bacterial enrichments in infected individuals, the chemical signals in root exudates could induce a decrease in species evenness in the rhizobacterial communities of infected plants, which may result in the similarity in diversities between infected and healthy plants. However, it is unclear whether this is the true case. Control experiments were needed to link the production and components of root exudates to the composition of bacterial communities.

### 4.2 Nematode infection reshaped the endophytic bacterial community

PPNs invade or pierce root cells to start their parasitism. Bacteria can take advantage of invading host roots through the wounds. However, the bacterial introduction accompanying nematode infection is not a random process (Bais et al., 2006; Li et al., 2023). In our study, significant taxonomic and functional shifts were presented in the endophytic bacterial communities in the nematode-parasitized organs (root knots) in *F. tikoua*. PCoA analysis separated the NRT (healthy roots) and IRK (root knots) samples well. Pairwise βNTI values for community differences of IRK vs. NRT and IRK vs. IRT were all greater than 2, indicating that the community dynamics in root knots were dominant by deterministic process, especially heterogenous selections (Stegen et al., 2012; Xun et al., 2019). Nematode endoparasitism induced not only structural modification (i.e., initiation of feeding sites, changing root architecture) but also physiological changes (i.e., triggering defense pathways) in host plants (Siddique and Grundler, 2018; Rutter et al., 2022). All these changes would alter selective pressures, providing deterministic environmental filtering to dilute or enrich given bacteria. The endophytic communities in root knots embraced significantly lower Shannon, ACE, and Chao indexes than those in healthy roots, suggesting the effects of environmental filters. Biological interactions can play important roles in the deterministic assembly process (Stegen et al., 2012; Xun et al., 2019). The cooccurrence networks of endobacteria in *F. tikoua* were consistent with the prediction. The co-occurrence network for the root-knot sample had fewer connected components but a higher average clustering coefficient, indicating more intensive bacterial interactions in root knots. All these results demonstrated that nematode infection reshaped the endophytic bacterial communities, and the effects were stronger than those on rhizosphere bacterial communities. Li et al. (2023) also tested a similar pattern in tomatoes.

The nematode infection induced the enrichment of special bacteria. Cooccurrence network analysis and genus composition tests revealed substantial enrichments of *Pseudomonas* in both infected roots and root knots and enrichments of *Streptomyces* in root knots. Members of *Pseudomonas* and *Streptomyces* have shown high antagonistic effects against plant pathogens. They employ diverse antagonistic mechanisms, such as producing broad-spectrum antibiotics, eliciting induced systemic resistance, and/or promoting the growth of host plants (Kerry, 2000; Pieterse et al., 2014; Pascale et al., 2020; Topalović et al., 2020). Some strains of *Pseudomonas* and *Streptomyces* have been used as biological control agents for PPNs (Tian et al., 2007; Köhl et al., 2019; Topalović et al., 2020). The enrichments of *Pseudomonas* and *Streptomyces* in nematode-infected plants suggested *F. tikoua* could actively recruit specific bacteria in response to nematode infection as other plants (see examples in Kerry, 2000; Topalović and Heuer, 2019; Topalović et al., 2020). However, FAPROTAX functional annotations did not detect significant differences in bacterial functions related to disease resistance among sample groups. It is still not clear whether it is due to low enrichment levels or other factors. The assembly and functions of root bacteria are highly context-dependent. A lot of factors can have strong effects on root bacterial communities, such as soil type, nutrient condition, and plant genotype (Fitzpatrick et al., 2018). More studies with controlled experiments, especially using metagenomic or metatranscriptomic approaches, were needed to further explore the effectiveness of these potentially beneficial bacteria in *F. tikoua*.

Nematode infection also induced the enrichment of nitrogen-fixing bacteria in infected individuals of *F. tikoua*. The relative frequency of *Allorhizobium–Neorhizobium–Pararhizobium–Rhizobium* was increased in infected roots as compared with healthy roots. Next, *Bradyrhizobium* was further enriched in root knots as compared with infected roots. Although no significant enhancement in nitrogen-fixing pathways was presented in infected roots, the enhancement did occur in root knots. The enrichments of nitrogen-fixing bacteria in nematode-parasitized organs were also found in other plants (Topalović and Vestergård, 2021; Li et al., 2023), suggesting the association between nitrogen-fixing bacteria with endoparasitic nematodes in host plants. Sedentary endoparasitic nematodes rely on host phloem sap for nutrient supply (Hoth et al., 2008; Bartlem et al., 2014; Siddique and Grundler, 2015). Plant phloem sap is usually poor in nitrogen, which would limit the performance of associated herbivores. It is believed bacteria related to nitrogen recycling or nitrogen-fixing have facilitated the survival and even the evolution of many phloem-feeding herbivores (Hansen and Moran, 2011; Sudakaran et al., 2017). It was suggested that plants could modulate the associated microbes to maximize their performance. It is paradoxical for infected plants to enrich nitrogen-fixing bacteria, which would support parasitic nematodes. How the enrichments happen is still unclear. It was revealed that some soil nematodes, such as *Caenorhabditis elegans*, could take nitrogen-fixing bacteria on their surfaces and in their guts (Horiuchi et al., 2005). It is worth exploring whether the parasitic nematodes also bring their own nitrogen-fixing bacteria with them to facilitate their parasitism in host plants.

### 4.3 From *Ficus* to field: suggestion for PPN controls

Nitrogen is essential for all organisms. The management of soil nitrogen supply has been applied to PPN controls (Akhtar and Malik, 2000). However, decreased nitrogen supply can also limit the growth of host plants. Documented studies found some soil nematodes fixing bacteria by themselves (Horiuchi et al., 2005). Our study and the study of Li et al. (2023) revealed nematode-infected individuals enriched nitrogen-fixing bacteria in parasitized organs. All these results suggested simple soil nutritional management could do more harm to host plants than to PPNs. A better understanding of the interaction among soil nitrogen-supplying bacteria, nitrogen-fixing bacteria, parasitizing nematodes, and host plants is critical for successful PPN control based on nitrogen management.

The antagonistic bacteria, such as *Pseudomonas* and *Streptomyces*, were also enriched in nematode-infected *F. tikoua* individuals. However, the enrichment of these antagonistic bacteria occurred in nematode-infected *F. tikoua* plants, but occurred in healthy tomato individuals (Tian et al., 2015). The different patterns indicate that plant–nematode–bacterium interactions are highly context-specific. A long history of nematodes in cultivated tomatoes may contribute to the enrichment of antagonistic endophytes. Tian et al. (2015) found *Pseudomonadales* to be among the most abundant groups in healthy tomato roots. Both experimental and field studies were needed to assess whether and how *Pseudomonas* and *Streptomyces* can be used to suppress PPNs in targeted plants.

## 5 Conclusion

Plant-associated bacteria play important roles in plant health. We explore the response of *F. tikoua* (a species with high application potential for fruit and medicine production) to nematode infections. Nematode infection had stronger effects on endophytic bacterial communities than on rhizosphere communities. The composition and structure of endophytic communities were reshaped by nematode infection, which can be attributed mostly to deterministic assembly processes. The infected plants selectively enriched nematode-antagonistic bacteria, which may help PPN suppression. Nitrogen-fixing bacteria were also enriched in the nematode-parasitized organs, which may facilitate the survival of PPNs. More studies are needed to explore how bacteria beneficial to both host plants and parasitic nematodes were enriched at the same time.

## Data availability statement

The original contributions presented in the study are publicly available. This data can be found at the National Center for Biotechnology Information (NCBI) using accession number PRJNA1065586.

## Author contributions

XM: Investigation, Data curation, Writing – original draft, Writing – review & editing. YG: Investigation, Writing – review & editing. LL: Investigation, Writing – review & editing. CL: Writing – review & editing. RW: Writing – review & editing. ML: Writing – review & editing. JD: Writing – review & editing. YC: Resources, Funding acquisition, Conceptualization, Writing – review & editing.
