# Supplementary material for: How the root bacterial community of Ficus tikoua responds to nematode infection: enrichments of nitrogen-fixing and nematode-antagonistic bacteria in the parasitized organs
Source: Front Plant Sci. 2024 Jun 28;15:1374431. doi: 10.3389/fpls.2024.1374431 (PMC11239514; doi:10.3389/fpls.2024.1374431)

Table S1. The nutrient contents in the soils and leaves of nematode-infected plants.

| Samples | Plant Group | pH | C (g·Kg^-1^) | N (g·Kg^-1^） | P (g·Kg^-1^） |
| --- | --- | --- | --- | --- | --- |
| Soil | Healthy | 8.25±0.10a | 8.78±1.22a | 1.38±0.083a | 0.070±0.006a |
|  | Infected | 8.26±0.14a | 7.08±1.00a | 1.30±0.12a | 0.067±0.005a |
| Leave | Healthy | / | 378.88±16.48a | 16.60±2.25a | 0.238±0.030a |
|  | Infected | / | 372.77±10.19b | 15.70±1.70a | 0.219±0.021a |

Table S2. The frequencies of top five bacterial phyla in rhizosphere soils and organs of healthy and nematode-infected *Ficus tikoua* plants. Sample groups: NRS, rhizosphere soil associated with healthy plant; IRS, rhizosphere soil associated with nematode-infected plants; NRT, root of healthy plant; IRT, root of infected plant; IRK, root knot (nematode-parasitized organ). The phyla which were dominant in all sample groups were highlighted in bold.

| Samples  Phyla | NRS | IRS | NRT | IRT | IRK |
| --- | --- | --- | --- | --- | --- |
| **Proteobacteria** (%) | 32.0 | 29.0 | 43.0 | 54.0 | 69.0 |
| **Actinobacteria** (%) | 23.0 | 23.0 | 21.0 | 16.0 | 20.0 |
| Acidobacteria (%) | 13.0 | 17.0 | 0.8 | 0.4 | 0.2 |
| **Chloroflexi** (%) | 10.0 | 10.0 | 5.0 | 7.0 | 4.0 |
| Bacteroidota (%) | 4.0 | 3.0 | 2.6 | 1.6 | 0.8 |
| Myxococcota (%) | 3.4 | 3.4 | 15.0 | 13.0 | 3.0 |
| Firmicutes (%) | 1.7 | 1.6 | 5.0 | 3.0 | 1.0 |
| sum (%) | 87.0 | 87.0 | 92.0 | 95.0 | 98.0 |

Table S3 The key indicators of the bacterial co-occurrence network on genus level.

| Sample | Number of Node | Number of Edge | Average Degree | Network Diameter | Average Clustering Coefficient | Connected Component | Modularity | Average Path Length |
| --- | --- | --- | --- | --- | --- | --- | --- | --- |
| NRT | 908 | 15158 | 33 | 6 | 0.758 | 6 | 0.787 | 2.973 |
| IRT | 908 | 16365 | 36 | 6 | 0.793 | 6 | 0.786 | 2.926 |
| IRK | 908 | 8109 | 17 | 5 | 0.769 | 5 | 0.777 | 2.939 |

Fig. S1 Paraffin cross-sections of *Ficus tikoua* root knots (a) stained with Hematoxylin and Eosin (HE) (b) stained with Safranin-Fast Green. N, nematode; *, feeding site (giant cell).


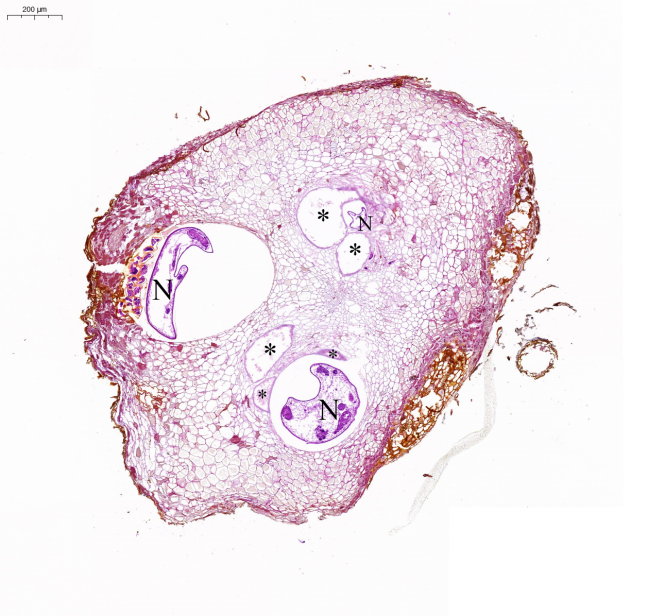

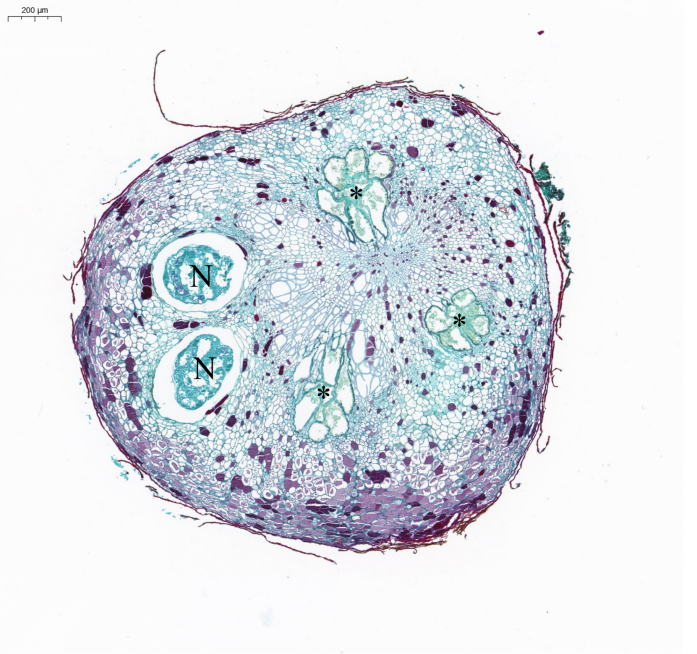

Supplement: Supplementary file 1 [file DataSheet_1.docx]
